# Protective CD8^+^ T-cell immunity to human malaria induced by chimpanzee adenovirus-MVA immunisation

**DOI:** 10.1038/ncomms3836

**Published:** 2013-11-28

**Authors:** Katie J. Ewer, Geraldine A. O’Hara, Christopher J. A. Duncan, Katharine A. Collins, Susanne H. Sheehy, Arturo Reyes-Sandoval, Anna L. Goodman, Nick J. Edwards, Sean C. Elias, Fenella D. Halstead, Rhea J. Longley, Rosalind Rowland, Ian D. Poulton, Simon J. Draper, Andrew M. Blagborough, Eleanor Berrie, Sarah Moyle, Nicola Williams, Loredana Siani, Antonella Folgori, Stefano Colloca, Robert E. Sinden, Alison M. Lawrie, Riccardo Cortese, Sarah C. Gilbert, Alfredo Nicosia, Adrian V. S. Hill

**Affiliations:** 1 The Jenner Institute Laboratories, University of Oxford, Old Road Campus Research Building, Roosevelt Drive, Oxford OX3 7DQ, UK; 2 Centre for Clinical Vaccinology and Tropical Medicine, University of Oxford, Churchill Hospital, Oxford OX3 7LJ, UK; 3 Division of Cell and Molecular Biology, Imperial College London, London SW7 2AZ, UK; 4 Clinical Biomanufacturing Facility, University of Oxford, Churchill Hospital, Oxford OX3 7JT, UK; 5 Centre for Statistics in Medicine, Linton Road, Oxford OX2 6UD, UK; 6 Okairos, viale Citta’ d’Europa 279, Rome 00144, Italy; 7 CEINGE, via Gaetano Salvatore 486, Naples 80145, Italy; 8 Department of Biochemistry and Medical Biotechnology, University of Naples Federico II, Via S. Pansini 5, Naples 80131, Italy; 9 These authors contributed equally to this work

## Abstract

Induction of antigen-specific CD8^+^ T cells offers the prospect of immunization against many infectious diseases, but no subunit vaccine has induced CD8^+^ T cells that correlate with efficacy in humans. Here we demonstrate that a replication-deficient chimpanzee adenovirus vector followed by a modified vaccinia virus Ankara booster induces exceptionally high frequency T-cell responses (median >2400 SFC/10^6^ peripheral blood mononuclear cells) to the liver-stage *Plasmodium falciparum* malaria antigen ME-TRAP. It induces sterile protective efficacy against heterologous strain sporozoites in three vaccinees (3/14, 21%), and delays time to patency through substantial reduction of liver-stage parasite burden in five more (5/14, 36%), *P*=0.008 compared with controls. The frequency of monofunctional interferon-γ-producing CD8^+^ T cells, but not antibodies, correlates with sterile protection and delay in time to patency (*P*_corrected_=0.005). Vaccine-induced CD8^+^ T cells provide protection against human malaria, suggesting that a major limitation of previous vaccination approaches has been the insufficient magnitude of induced T cells.

## Abstract (web summary)

Induction of protective immunity mediated by CD8^+^ T cells has been a long sought goal in vaccinology. Here, Ewer *et al.* report induction of protective efficacy against *Plasmodium falciparum* malaria in a phase IIa prime-boost vaccine trial where efficacy correlates strongly with induced CD8 T-cell responses.

## Graphical abstract

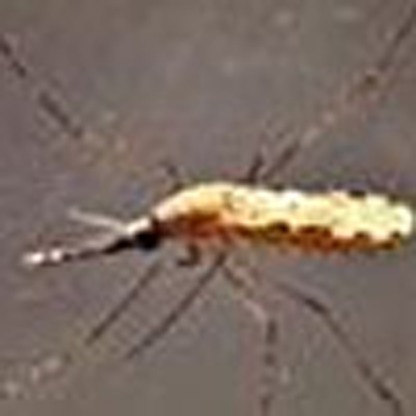

A major goal of modern vaccinology is to extend the capacity of current vaccines to include provision of protective immunity mediated by CD8^+^ T cells^1^. CD8^+^ T cells provide protective immunity against a wide range of infectious pathogens such as HIV, influenza virus and CMV and some tumours, in many animal species and humans, suggesting that induction of CD8^+^ T cells by immunization could provide prophylactic or even therapeutic efficacy against a wide range of pathogens, particularly those that survive inside cells^2^,^3^,^4^,^5^,^6^. The broader range of expression of HLA class I molecules, on almost all nucleated cells, makes CD8^+^ T cells more desirable effectors than CD4^+^ T cells, which can target only HLA class II-positive cells, and CD8^+^ T cell memory is generally very durable^7^. However, despite substantial efforts to harness cellular immunity as an effector mechanism for a new generation of T–cell-based vaccines, almost all licensed vaccines provide protection that appears to be primarily mediated by antibodies. The sole exception, the BCG vaccine against tuberculosis, appears to protect primarily by induction of CD4^+^ rather than CD8^+^ T cells^8^.

A wide variety of vaccine technologies have been found to provide CD8^+^ T-cell-dependent protection in small animal models of infection and cancer but translating this to humans has been problematic. It remains unclear whether the main problem is induction of a CD8^+^ T cell response of the wrong quality^9^ or insufficient magnitude^10^. Interestingly, the levels of CD8^+^ T cell responses inducible in mice are typically at least an order of magnitude greater than those in humans^11^, and rare examples of T-cell-mediated efficacy in non-human primates^12^ have induced levels of CD8^+^ T-cell response higher than those reported in almost all clinical trials.

CD8^+^ T cells targeting the liver stage of malaria infection are protective in animal models and can be induced by both irradiated sporozoites and several subunit vaccines^13^. Several antigens including thrombospondin-related adhesion protein (TRAP)^14^ are protective. A particularly effective means of inducing CD8^+^ T cells was discovered in murine malaria models whereby two replicating^15^ or non-replicating^11^,^14^,^16^ viral vectors were found to induce CD8^+^ T-cell mediated immunity provided that a poxvirus vector was used as the boosting agent.

In human clinical trials we have found that such prime-boost approaches using either plasmid DNA or a fowlpox vector to prime immune responses and the replication-deficient modified virus Ankara (MVA) strain of vaccinia as a boosting vector could induce modest protective efficacy, manifested mainly as delay in time to patency, against controlled human malaria infection (CHMI) with a well-defined five-bite sporozoite challenge protocol^17^,^18^. However, this efficacy was associated with CD4^+^ T-cell responses while induction of CD8^+^ T cells was very weak. More effective priming agents for induction of CD8^+^ T cells in pre-clinical studies are human^16^ or, more recently identified, chimpanzee-derived^11^,^19^ adenoviruses.

The induction of higher-level protective efficacy with vectors is a priority for malaria vaccine development as only one malaria vaccine has induced repeatable efficacy in field trials^20^,^21^; this protective efficacy is associated with high titres of antibodies against sporozoites but no CD8^+^ T cell induction. Addition of a partially effective liver-stage component to this anti-sporozoite vaccine might provide substantially higher efficacy^22^.

We assess here the efficacy of chimpanzee-derived simian adenovirus 63 (ChAd63, previously denoted AdCh63) expressing the ME-TRAP insert^17^,^18^,^23^ with MVA expressing the same insert (MVA ME-TRAP) in a heterologous prime-boost regimen. This open label phase IIa sporozoite (CHMI) study followed successful pre-clinical studies in mice^11^ and rhesus macaques^24^ and a phase I dose escalation study^23^ that showed adequate safety and exceptional T-cell immunogenicity. We demonstrate that using this approach, protective efficacy against heterologous strain sporozoite CHMI is observed in more than half of the prime-boost vaccinees (*n*=14, *P*=0.008, log-rank Mantel Cox test), and protection correlates with antigen-specific interferon gamma (IFNγ)-producing CD8^+^ T cells (*P*<0.001, two-tailed Spearman’s correlation), but not with antibodies.

## Results

### Study design and vaccine safety

We conducted the efficacy study in two parts (challenges A and B) (Supplementary Fig. S1a). We first compared this adenovirus prime-MVA boost regimen, using one dose of each vaccine 8 weeks apart (*n*=8), with a single dose of the adenovirus vector used alone (*n*=10) with unvaccinated controls (*n*=6) in challenge A, and then repeated the comparison between the prime-boost regimen (*n*=6) and controls in challenge B to confirm our findings. We challenged prime-boost vaccinees 2–3 weeks (range 14–21 days) following MVA vaccination (day 70–77 post-ChAd63), as before^17^,^18^, and adenovirus-alone volunteers 28 days (range 21–28) following priming vaccination for optimal comparability, as this encompassed the period of peak immunogenicity identified in a phase I study^23^. Both vaccines were well tolerated (Supplementary Fig. S1b,c).

### Cellular and humoral immunity induced by vaccination

**Figure 1:**
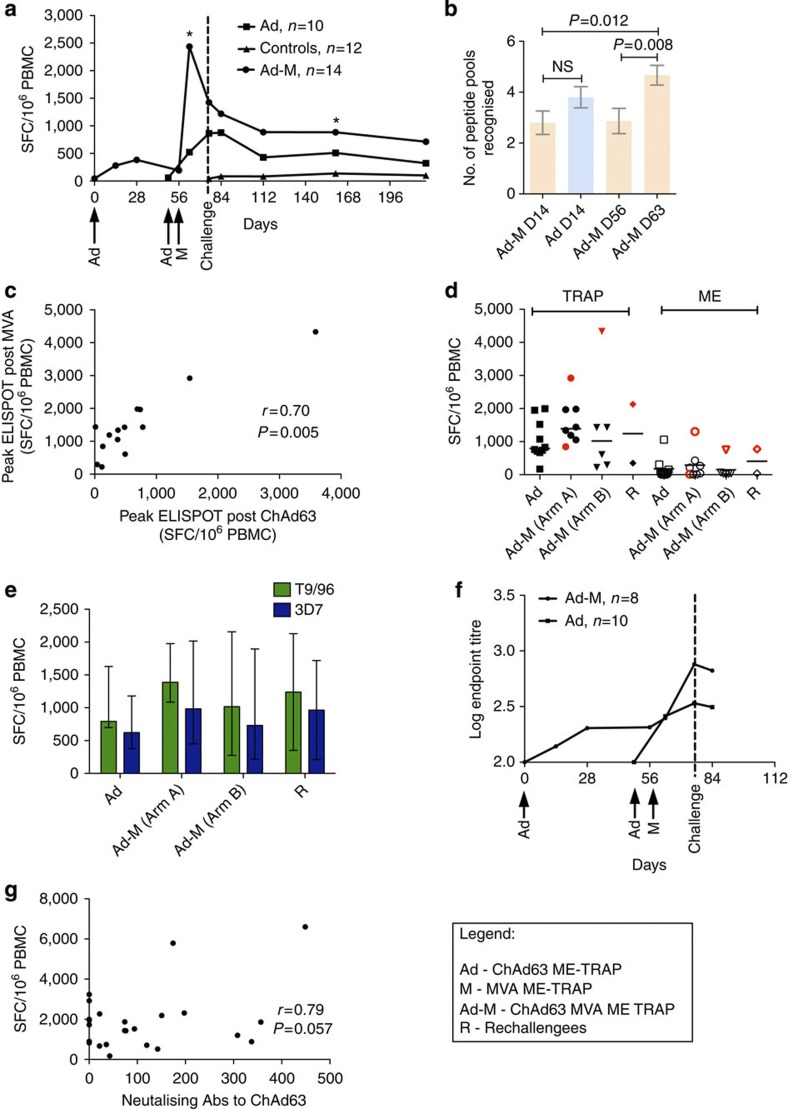
Immunogenicity of prime-boost vaccination with ChAd63-MVA ME-TRAP. (**a**) Median ME-TRAP IFNγ *ex vivo* ELISPOT responses to the T9/96 strain for each group, *P*=0.04 for peak immunogenicity (D28 for A (*n*=10) and D63 for AM (*n*=14)), *P=0.012 for difference in immunogenicity at D166, both two-tailed Mann–Whitney. (**b**) Breadth of TRAP-specific ELISPOT responses before (D14, D56) and after boosting (D63) with MVA ME-TRAP, using T9/96 strain peptides, showing mean (±s.e.m.) number of pools recognized. (**c**) Correlation of peak ELISPOT responses to T9/96 TRAP post-prime and at time of sporozoite challenge. (**d**) Individual responses to TRAP and ME at time of sporozoite challenge (data points in red represent sterilely protected volunteers). (**e**) Median with IQR IFNγ ELISPOT responses to TRAP peptides from the heterologous vaccine and challenge strains of *P. falciparum*. (**f**) IgG antibodies to TRAP boosted by MVA compared with ChAd63 alone (medians); differences not statistically significant. (**g**) Spearman’s correlation of pre-existing antibodies to ChAd63 with peak vaccine-induced ELISPOT responses to TRAP (T9/96 strain).

Immune responses to ME-TRAP in the prime-boost vaccinated group (ChAd63-MVA) peaked 1 week after boosting at a median of 2,436 (inter-quartile range (IQR): 1,064–3,862) spot-forming cells (SFC) per million peripheral blood mononuclear cells (PBMC) in an *ex vivo* IFNγ enzyme-linked immunospot (ELISPOT) assay, compared with a median of 864 SFC/10^6^ PBMC (IQR: 710–1,910) in the prime-only group (ChAd63) (*P*=0.04, two-tailed Mann–Whitney; Fig. 1a). Follow-up of all vaccinees to day 150 post challenge showed good maintenance of effector T-cell frequencies with responses in the prime-boost group of 712 SFC/10^6^ PBMC (IQR: 310–1,412) representing 50% of the response at the time of challenge. This level of immunogenicity is considerably higher than that found with previous prime-boost regimes with the same antigenic insert^17^,^18^, and also appears to be higher than in all reported studies of vectored vaccines for HIV^25^ and malignant diseases^26^. We observed ELISPOT responses to TRAP in 100% of vaccinated volunteers and multiple peptide pools (>3/6) were recognized in every case, both at the peak of the response and the time of CHMI. Epitope mapping with individual 20mer peptides identified at least 16 different potential determinants. At time of challenge in part B, mapping with individual 20mer peptides showed an average of 7.5 peptides recognized per volunteer (range 3–18), although the use of overlapping peptides means that the number of actual epitopes may be as much as 33% lower if an epitope was represented in two adjacent pools. Boosting with MVA significantly increased the breadth of the response in the prime-boost group as measured by an increase in the number of peptide pools recognized before and after MVA (*P*=0.012; two-tailed Mann–Whitney test; Fig. 1b), and the magnitude of the ELISPOT response to TRAP after adenovirus priming was strongly associated with the subsequent response to MVA (*r*_s_=0.70, *P*=0.005 (two-tailed Spearman’s correlation); Fig. 1c). T-cell responses targeted predominantly the TRAP antigen rather than the ME string (Fig. 1d). Responses to the heterologous 3D7 challenge strain antigen were on average 73% of the response to the vaccine strain antigen, T9/96 (Fig. 1e). Vaccination with ChAd63 ME-TRAP also induced modest detectable IgG antibody responses to TRAP in 16/18 volunteers, and these levels were boosted twofold (17/18 responders) after MVA ME-TRAP (measured in challenge A only; Fig. 1f) to a geometric mean endpoint titre of 250. Neutralizing antibody (nAb) titres to the adenovirus vector measured pre-vaccination in challenge A were generally low as expected for a simian adenovirus (median titre 74, IQR: 0–168) and these antibody levels did not correlate negatively with the magnitude of vaccine-induced T cell or antibody responses to TRAP, but a trend (*r*_*s*_=0.79 *P*=0.057, two-tailed Spearman’s correlation) towards higher induced peak T cell responses to the insert was noted in those with antibodies to the vector (Fig. 1g). In view of this lack of impact of nAb titre on immunogenicity, we removed the exclusion criterion of nAb titre >200 for vaccinated volunteers in challenge B and observed no attenuation of immunogenicity, nor was there any relationship between nAb titre and time to patency by blood film.

### Protective efficacy of ChAd63-MVA ME-TRAP against CHMI

Overall, we exposed 24 vaccinees and 12 unvaccinated control volunteers (6 in each challenge) to five infectious *A. stephensi* mosquito bites in a standard procedure used in over 1,300 vaccine trial subjects worldwide including >300 at this centre (Supplementary Fig. S1a). The TRAP antigen in the infecting strain (3D7) differs by 37 amino acids (~6.5%) from the vaccine strain (T9/96), thereby constituting a heterologous strain challenge^17^. As expected, all 12 non-immunized controls developed malaria, as did all 10 vaccinees who received only a single adenovirus immunization. Of the 14 prime-boost vaccinees, 3 (21.4%, 95% CI: 46.0–3.2%) were sterilely protected, 2/8 in challenge A and 1/6 in challenge B, a statistically significant protection rate, which is higher than in all previous trials with other vectored vaccine regimes (0–12.5%)^17^,^18^,^27^,^28^.

**Figure 2:**
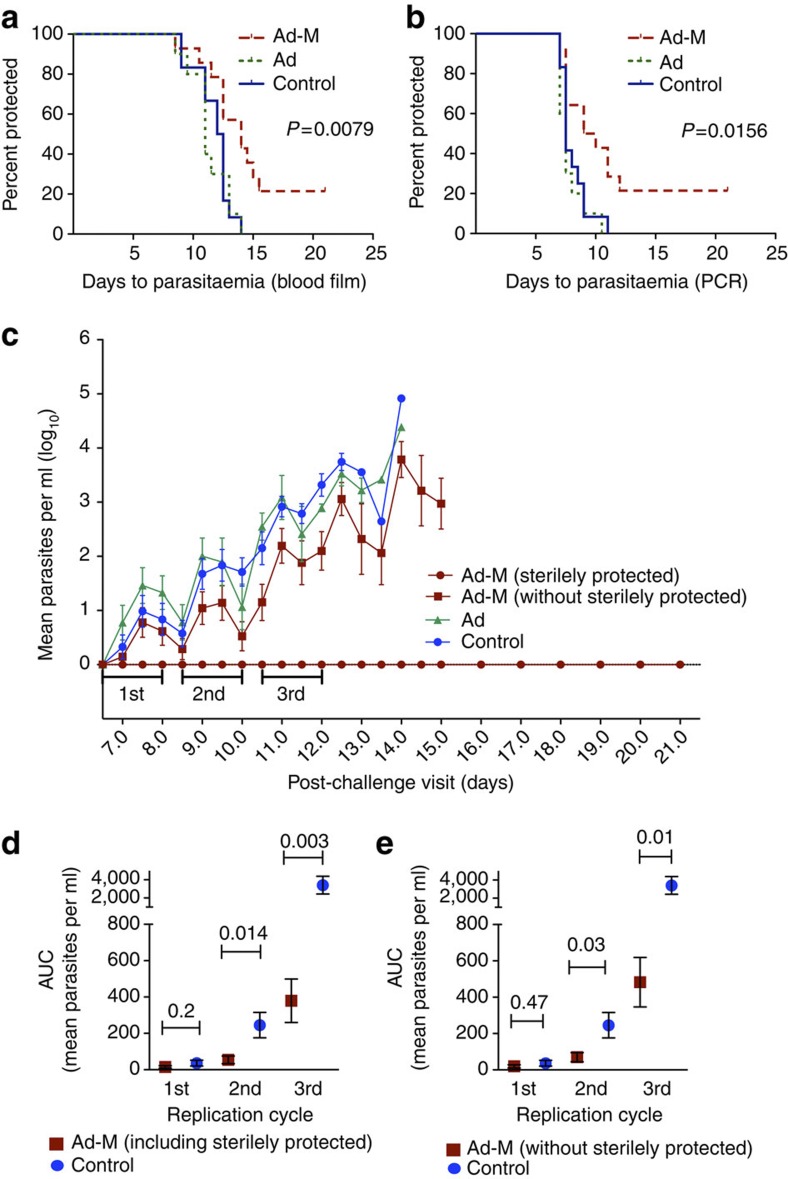
Vaccine efficacy assessments by time to microscopic patency and PCR measures. Kaplan–Meier log-rank comparison of days to positive blood film (**a**) and >20 parasites per ml by PCR (**b**) between adenovirus-MVA prime-boost (Ad-M) group (*n*=14), adenovirus-only (Ad) group (*n*=10) and controls (*n*=12). Mean days to positivity by blood film: Ad-M 14.6 days (95% CI: 12.3–16.8), adenovirus-alone group (Ad) 11.3 days (95% CI: 10.2–12.5), control group 11.8 days (95% CI: 10.8–12.7); and to 20 parasites per ml by PCR: Ad-M 11.6 days (95% CI: 8.5–14.7), Ad 7.8 days (95% CI: 7.0–8.6), control group 8.1 days (95% CI: 4–8.8). (**c**) Group mean log-transformed PCR data (error bars represent s.e.m.). Area under curve analysis of parasite densities comparing controls to vaccinees (either including (**d**) or excluding (**e**) volunteers that were sterilely protected) at days 6.5–8 (first cycle post hepatocyte release), days 8.5–10 (second cycle) and 10.5–12 (third cycle) post challenge. Over the second and third cycles, there is a significant reduction in vaccinees’ parasite densities compared with controls; the lack of significance at cycle one probably reflects low power due to very low parasite densities. Comparison of areas under curves for all three cycles combined also shows a significant reduction in parasite densities between vaccinees and controls (*P*=0.003 when including sterilely protected vaccinees, *P*=0.01 when excluding sterilely protected vaccinees, (two-tailed *t*-test).

In addition five prime-boost vaccinees (36%, 95% CI: 64.4, 7.0%) developed malaria at day 14 or later, >2 days later than unvaccinated control volunteers (2/8 challenge A, 3/6 challenge B), a significant delay to patent parasitaemia indicating a strong vaccine-related biological impact on liver-stage parasites comparing the five delayed vaccinees (14.6 days) to the control group (11.8 days). Based on the 2.8 day difference in mean time to parasitaemia, at a 12-fold parasite growth rate per 48 h^29^, there is a 27-fold reduction in parasite density emerging from the liver corresponding to a 96% reduction in liver parasite burden. Kaplan–Meier survival analysis of time to parasitaemia, the primary efficacy endpoint of the trial, demonstrated significant delay in time to patent parasitaemia in the prime-boost group compared with the unvaccinated control group as measured by blood film microscopy (*P*=0.008, log-rank test, Fig. 2a). There were no significant differences in time to patency between the two challenges for either prime-boost (*P*=0.61) or control volunteers (*P*=0.14, both log-rank test).

This delay to patent parasitaemia among prime-boost vaccinees was also evident using a sensitive real-time quantitative PCR assay of blood parasite densities^30^ to measure time to >20 parasites per ml as an endpoint (*P*=0.016, log-rank test, Fig. 2b, raw data shown in Supplementary Table S1). The clinical impact of prime-boost was also reflected by group parasite growth dynamics, with a significant reduction in mean parasite density measured by PCR over the second and third parasite replication cycles in Ad-M recipients compared with controls (*P*=0.014 and 0.0003 two-tailed Mann–Whitney or *t*-test for area under curve; Fig. 2c,d). This vaccine-induced effect remained when sterilely protected vaccinees were removed from the analysis (*P*=0.03 and 0.01 two-tailed *t*-test for area under curve during second and third replication cycles, respectively; Fig. 2c,e). The area under the curve of parasite density over the first three replication cycles in infected volunteers was significantly negatively correlated with time to blood-film diagnosis (*r*_s_=−0.63, *P*<0.002, two-tailed Spearman’s correlation). Therefore, this substantial difference in parasite densities between infected vaccinees and controls at days 8–12 post challenge, shortly after parasites enter the blood from the liver, provides further evidence of a significant vaccine effect.

We re-challenged both sterilely protected vaccinees from challenge A 8 months after the first sporozoite exposure: one volunteer was sterilely protected again, and one delayed by 48 h when compared with the controls (*P*=0.034, log-rank test); the sterilely protected vaccinee from challenge B also showed a significant delay to patency on re-challenge (day 14.0 compared with a mean of 10.2 days for the controls in that challenge) after an 8-month interval, indicating some maintenance of protective immunity (overall *P*=0.0047, log-rank test, for the 3 re-challengees compared with 12 controls).

### Assessment of vaccine-induced correlates of protection

**Figure 3:**
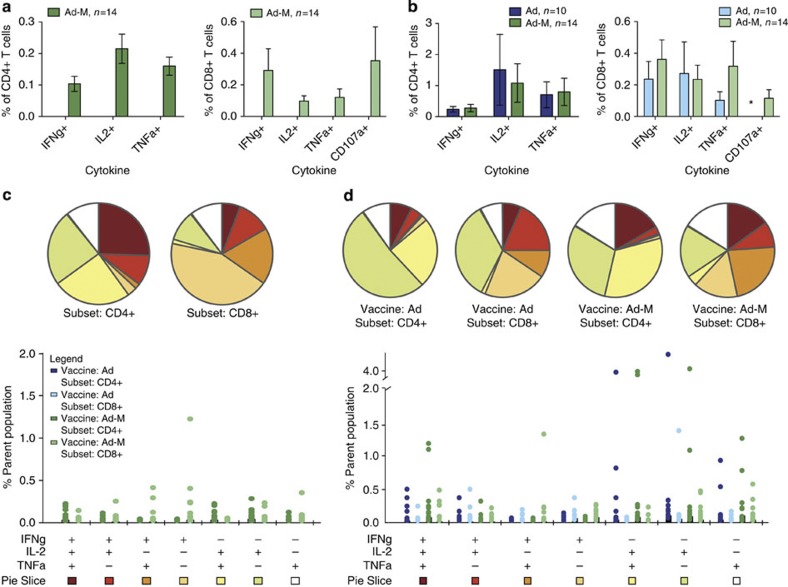
Functionality of TRAP-specific CD4^+^ and CD8^+^ T cells. (**a**) Peak immunogenicity (day 63=7 days after boosting) and (**b**) time of challenge (day 76). *N*=10 for adenovirus-only group and *n*=14 for Ad-M group. Frequencies of cytokine-secreting CD4^+^ and CD8^+^ T cells. *Staining for CD107a^+^ expression performed at challenge B only. Mean (with s.e.m.) responses are shown. (**c**,**d**) T cell responses shown are grouped according to number of functions at peak (**c**) and challenge (**d**). Pie charts summarize the fractions of the total response that are positive for three, two or one functions. All possible combinations of functions are shown in the bar chart stratified by vaccination regimen, with the *y* axis showing the percentage of CD4^+^ or CD8^+^ cells. Data points represent individual volunteers.

Analysis of immune responses by flow cytometry showed that vaccination induced high frequencies of TRAP-specific CD4^+^ and CD8^+^ T cells (Fig. 3a,b), containing IFNγ, interleukin-2 (IL-2), tumour necrosis factor-alpha (TNFα) or displaying CD107a, a marker of the capacity of T cells for cytotoxic degranulation. A sample gating strategy is shown in Supplementary Fig. S2. T cells induced by the prime-boost regimen were more polyfunctional than those induced by vaccination with ChAd63 alone (Fig. 3c,d). The median frequency of polyfunctional CD4^+^ T cells containing IFNγ, IL-2 or TNFα simultaneously at the time of challenge in the prime-boost group was 0.1% of antigen-specific CD4^+^ T cells (IQR: 0.02–0.24) compared with 0.04% (IQR: 0.02–0.25) for the adenovirus-only group. Frequencies of polyfunctional CD8^+^ T cells were also higher in the prime-boost group (median 0.03%, IQR: 0–0.24) compared with 0.01% (IQR: 0–0.03) in the adenovirus-alone group. However, the total percentage of CD8^+^ T cells containing IFNγ in the prime-boost group was much higher (median: 0.12% (IQR 0.05–0.7); mean: 0.36% (s.e.m. 0.12)), representing exceptionally strong immunogenicity.

**Figure 4:**
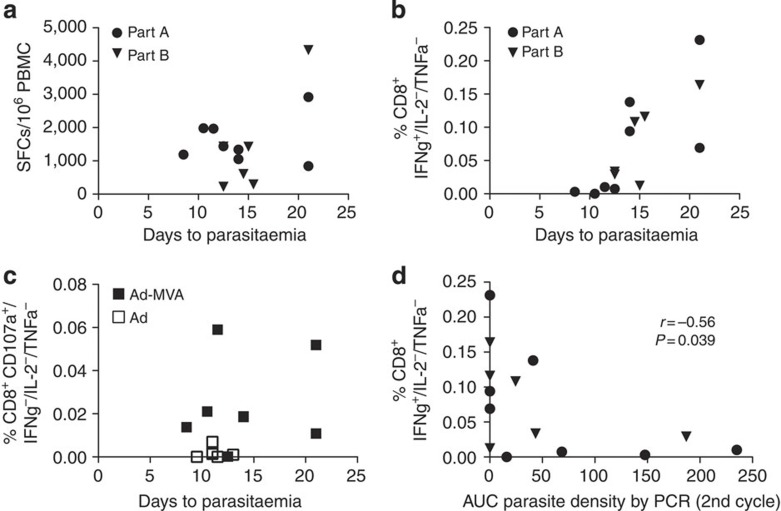
Correlates of protective efficacy. (**a**) Correlation of time to parasitaemia with *ex vivo* ELISPOT responses for Ad-MVA vaccinees, *P*=0.97. (**b**) Correlation of time to parasitaemia with frequency of CD8^+^ IFNγ^+^/IL-2^−^ /TNFα^−^ for Ad-MVA vaccinees, *P*=0.0005. For vaccinees receiving Ad alone or Ad-MVA (*n*=24), correlation of time to parasitaemia with frequency of CD8^+^ IFNγ^+^/IL-2^−^ /TNFα^−^, *r*_s_=0.61, *P*=0.002. Both (**a**) and (**b**) were assessed at the time of sporozoite challenge. (**c**) Correlation of time to parasitaemia with frequency of CD107a^+^/ IFNγ^−^/IL-2^−^/TNFα^−^ CD8^+^ T cells at day 150 post challenge in challenge A, *P*=0.02. (**d**) Correlation of parasite density in second replication cycle with frequency CD8^+^ IFNγ^+^/IL-2^−^ /TNFα^−^ for Ad-MVA vaccinees. All correlations were performed using two-tailed Spearman’s correlation.

Both cellular and antibody responses at the time of challenge were studied for their association with time to patent parasitaemia. *Ex vivo* and cultured IFNγ ELISPOT responses to TRAP (summed across the pools of peptides representing the T9/96 strain antigen) showed no statistically significant association with time to patency (Fig. 4a and Table 1). Antibodies to TRAP were measured by ELISA in part A of the trial and did not correlate with vaccine performance, so we focused on cellular immune correlates in part B.

Further analysis of immune correlates of vaccine performance included measures of polyfunctional as well as monofunctional CD4^+^ and CD8^+^ T cells and mean fluorescence intensity (geometric and integrated). In part A, we analysed combined data from all vaccinees and identified the frequency of CD8^+^ T cells secreting IFNγ, but not IL-2 or TNFα, as the strongest correlate of time to patency (*r*_s_=0.63, *P*=0.005, two-tailed Spearman’s correlation; Fig. 4b and Table 1). In the prime-boost group alone, the association was also evident despite smaller numbers (*r*_s_=0.84, *P*=0.011, two-tailed Spearman’s correlation). However, cytokine production on a per-cell basis was not associated (Table 1), suggesting that the quantity of cytokine secreted alone was not a key protective factor. This observation contrasts with findings in murine leishmaniasis where polyfunctional CD4^+^ T cells and quantity of cytokine secreted per cell were predictive of protective vaccine efficacy^9^, but is consistent with our recent data in a murine malaria model^11^. Analysis of CD107a^+^ expression at a later time point (150 days after challenge A) showed that the frequency of these lytic CD8^+^ T cells also correlated with efficacy (*r*_s_=0.61, *P*=0.02, two-tailed Spearman’s correlation; Fig. 4c). We then reassessed and confirmed the association between time to patency and CD8^+^ T cells secreting IFNγ alone in prime-boost vaccinees in challenge B (*r*_s_=0.64, *P*=0.018, two-tailed Spearman’s correlation). Analysis of the combined data from challenges A and B showed a very clear correlation both with time to patency (*r*_s_=0.81, *P*=0.0005; Table 1) and PCR-quantified early blood parasite density (*r*_s_=−0.56, *P*<0.04, two-tailed Spearman’s correlation, Fig. 4d). A secondary analysis of subgroups showed that IFNγ-secreting CD8^+^ T cells were also significantly higher both in vaccinees showing sterile (*P*<0.01) protection and also in those showing only a delay in time to patent parasitaemia (*P*<0.05) compared with other non-protected vaccinees (one-way ANOVA with Bonferroni’s multiple comparison test, Supplementary Fig. S3). Thus, the level of IFNγ-secreting monofunctional CD8^+^ T cells induced by vaccination effectively stratified the degree of impact on the parasitaemia observed, from complete liver clearance (sterile efficacy) to substantial but incomplete clearance (reflected in a delay to patency), and to no detectable effect.

## Discussion

Heterologous prime-boost immunization regimes have provided a means of inducing strong T-cell immunity in pre-clinical models. A priming immunization with a chimpanzee adenoviral vector followed by an MVA boost here induced durable responses of exceptional magnitude with a high proportion of cytokine-secreting CD8^+^ T cells. The overall level of T-cell response induced here is 5–10 fold higher than with previous prime-boost regimes using the same antigenic insert^17^,^18^, with an equal proportion of cytokine secreted from CD4^+^ and CD8^+^ T cells, and much higher antigen-specific CD8^+^ T-cell responses than those induced by DNA vaccines or irradiated sporozoites^31^,^32^. Even without the MVA boost, the adenovirus-primed responses here appear stronger than those reported with human adenoviral vectors^33^ and this may in part reflect the very low level of anti-vector immunity to simian vectors in humans^34^,^35^.

Two levels of pre-erythrocytic malaria vaccine performance may be evaluated in phase IIa CHMI trials: sterile efficacy where all parasites are cleared at the pre-erythrocytic stages or delayed patency^17^,^18^,^36^,^37^, or partial efficacy, which is observed when sufficient parasites are cleared to produce a 2-day delay in time to patency, corresponding to clearance of an estimated >95% of pre-erythrocytic parasites^29^. Vaccinees showing delay in time to patency have a substantially reduced parasite density in their blood shortly after liver to blood infection, as shown by sensitive PCR analyses (Supplementary Fig. S3 and Supplementary Table S1). Three vaccinees here showed sterile efficacy and five showed delay totalling 8/14 (58%) with ChAd63-MVA, compared with 9/38 individuals (24%, *P*=0.02, *χ*^2^ test) with earlier DNA–MVA and FP9–MVA regimes that used the same ME-TRAP malaria insert^17^,^18^,^28^. In contrast, no sterile efficacy or delay in time to patency was observed in those administered the ChAd63 vector without an MVA boost, underscoring the importance of heterologous prime-boost immunization regimes for potent CD8^+^ T-cell induction.

We show that CD8^+^ T cell levels comparable to those achieved in macaque pre-clinical models^24^,^38^ can now be induced in humans with a significant correlation of CD8^+^ T-cell immunogenicity with efficacy. We show elsewhere that comparable very potent immunogenicity can be induced with these vaccine vectors using two other malaria inserts^39^,^40^, and similar results with antigens from HCV^41^ suggest that this new prime-boost approach may be widely applicable. Although several T-cell subpopulations were weakly correlated with protection in the first arm, when the study was repeated, thereby increasing the overall sample size, the weaker correlations became non-significant, with only the frequency of monofunctional IFNγ-secreting CD8^+^ T cells showing a significant association with protection when data from the two arms of the study were combined. In this and previous studies^23^,^36^,^37^ the greater magnitude of T-cell immunogenicity induced by ChAd-MVA heterologous prime-boost immunization correlates with an increase in the number of detectable epitopes recognized so it is likely that increased breadth also correlates with efficacy, and distinguishing between these two will require larger studies.

Importantly, the levels of CD8^+^ T-cell response required for efficacy identified here, though substantial, are much lower than the extremely high CD8^+^ T-cell levels required for efficacy in murine malaria models, possibly in part related to the longer duration of the liver stage, typically 7 days versus 2 days^10^,^11^, with *P. falciparum* than rodent *Plasmodia spp*. Interestingly, the protective value of monofunctional IFNγ-secreting T cells, shown here in humans, is consistent with our recent murine malaria data^11^.

To our knowledge a CD8^+^ T cell correlate of vaccine-induced protection has not previously been reported in humans, although there is evidence that CD4^+^ T-cell responses are relevant for efficacy of tuberculosis and possibly other vaccines^42^. These results also suggest that single-vector immunization strategies assessed previously in other diseases may simply have been too weakly immunogenic to reach the threshold level needed for significant efficacy.

The leading candidate malaria vaccine, RTS,S/AS01, does not induce malaria-specific CD8^+^ T cells and appears to work primarily through antibody induction against sporozoites, preventing them entering liver cells^43^. We report here the second highest level of statistically significant efficacy amongst >30 malaria vaccine candidates assessed in phase II trials, exceeded only by RTS,S, which is currently in a large phase III trial in Africa. Data from that trial recently showed an efficacy of just 31% against clinical malaria when used in the key target population of 6–12-week-old infants, possibly related to lower than expected antibody immunogenicity in this age group^20^,^21^, despite RTS,S having previously demonstrated 50% sterile efficacy in adults against CHMI^37^. It appears unlikely that RTS,S used alone will offer a cost-effective vaccine of high efficacy for deployment, at least in the key target age group of young infants^20^. However, importantly, combining partially effective antibody and CD8^+^ T-cell-inducing vaccines leads to synergistic efficacy in murine malaria^22^.

The very high efficacy levels observed pre-clinically with such a combination approach are probably achieved by allowing T cells to clear the liver of parasitized hepatocytes more effectively after a partially effective anti-sporozoite vaccine has reduced sporozoite and thereby infected hepatocyte numbers. Estimates of the tiny numbers of sporozoites that survive the antibodies induced by RTS,S to enter liver cells in human vaccinees^29^ further emphasize that CD8^+^ T-cell-inducing vaccines should be more effective in combination with anti-sporozoite vaccines. Hence, our observation of high level but often incomplete clearance of pre-erythrocytic parasites in 8/14 vaccinees with a liver-stage vaccine suggests that this vectored vaccine approach could be particularly effective if combined with an additional component, such as RTS,S, that targets sporozoites with protective antibodies. So, we report here what could be a key component for a highly efficacious vaccine against malaria. Assessment of the efficacy and deployability of such combination vaccine approaches is now a priority for malaria control strategies, particularly as both approaches are currently in trials in African infants^21^,^44^.

More generally, the feasibility of inducing protective CD8^+^ T-cell immunity in humans using safe, readily manufactured, non-replicating viral vectors offers the prospect of assessing potent CD8^+^ T-cell-based vaccines in humans for prophylaxis and immunotherapy of many infectious and non-infectious diseases.

## Methods

### Clinical trial design

We conducted the study at Centre for Clinical Vaccinology and Tropical Medicine and Vaccinology, University of Oxford, with the CHMI procedure performed at Imperial College, London. We recruited healthy malaria naïve adults aged 18–50 years old from the Oxford area during Feb 2009 to Jan 2010 (inclusion and exclusion criteria are described in Supplementary Methods). All volunteers gave written informed consent prior to participation and the study was conducted according to the principles of the Declaration of Helsinki and in accordance with Good Clinical Practice (GCP). Approvals were granted by the Oxfordshire Research Ethics Committee (OXREC A 09/HO604/9) and the Medicines and Healthcare products Regulatory Agency (EudraCT 2008-006804-46). The trial was registered with ClinicalTrials.gov number NCT00890760. The Clinical Biomanufacturing Facility, University of Oxford (ChAd63) and IDT Biologika, Rosslau, Germany (MVA), manufactured study vaccines under Good Manufacturing Practice conditions. A Local Safety Monitor provided safety oversight and GCP compliance was externally monitored.

We vaccinated 15 volunteers with ChAd63 ME-TRAP 5 × 10^10^ v.p. intramuscularly followed by MVA ME-TRAP 2 × 10^8^ p.f.u. intradermally 56 days later. We vaccinated another group of 10 volunteers with a single dose of ChAd63 ME-TRAP 5 × 10^10^ v.p. intramuscularly. Group allocation was non-randomized. Volunteers attended clinical follow-up at days 2, 14 and 28 following ChAd63 ME-TRAP, and at days 56, 63 and 76 following MVA ME-TRAP. Safety assessment including blood sampling for safety was conducted as previously described^17^,^18^,^23^ (adverse event assessment criteria are shown in Supplementary Tables S4–S7). One volunteer in the prime-boost group in CHMI A was unable to proceed to challenge having moved from the study area.

Five *Anopheles stephensi* mosquitoes, each with 10^2^–10^4^ sporozoites per salivary gland, were allowed to bite each subject, thus delivering 3D7 strain *P. falciparum* sporozoites, 14–21 days after the final vaccination. This procedure took place over 2 days with the same number of vaccinees and controls exposed each day. Monitoring took place twice daily by using Giemsa-stained thick blood films, which were considered positive if a single morphologically correct parasite was seen, and by quantitative PCR starting on day 6.5 until day 14 and then once daily until the end of the study period at day 21. Subjects were treated with Riamet after the first confirmed positive blood film or at day 21 if no parasitemia was detected. In addition to vaccinated subjects, six unvaccinated subjects were infected with malaria as infectivity controls. We reviewed volunteers at 35, 90 and 150 days following challenge for safety and immunology assessment.

Sporozoite CHMI group sizes are typically small but power to detect differences between the vaccinees and the controls is improved by the use of Kaplan–Meier analysis of time to patent parasitaemia. Partially effective vaccines should delay average time to parasitaemia in non-sterilely protected individuals if they protect any number of volunteers fully. The primary endpoint analysis was time to patent parasitaemia for the vaccine groups (prime-boost (*n*=14) and ChAd63-alone (*n*=10)) compared with unvaccinated controls (*n*=12) by Kaplan–Meier analysis (two-tailed). Significance testing used the log-rank test.

### Immunological and molecular assays

*Ex vivo* (18-h stimulation) and cultured ELISPOT (10-day stimulation) assays were performed using Multiscreen IP ELISPOT plates (Millipore), human IFNγ SA-ALP antibody kits (Mabtech) and BCIP NBT-plus chromogenic substrate (Moss Inc). Cells were cultured in RPMI (Sigma) containing 10% heat-inactivated, sterile-filtered fetal calf serum, previously screened for low reactivity (Labtech International). Antigens were tested in duplicate with 250,000 PBMC added to each well of the *ex vivo* ELISPOT plate^18^ and 100,000 cultured T cells in the cultured ELISPOT assay. TRAP peptides were 20 amino acids in length, overlapping by 10 amino acids (Neopeptide), assayed in 6 pools of 7–10 peptides at 10 μg ml^−1^. Responses were averaged across duplicates, responses in unstimulated (negative control) wells were subtracted and then responses in individual pools were summed for each strain of the TRAP antigen. ME responses were assayed in a single pool and peptide pool configurations are shown in Supplementary Tables S2 and S3. Staphylococcal enzyme B (0.04 μg ml^−1^) and phytohaemmagglutinin-L (20 μg ml^−1^) were used as a positive control. Epitope mapping was performed using individual 20mer peptides spanning the length of the T9/96 TRAP protein in single ELISPOT wells, each containing 100,000 PBMC. Plates were counted using an AID automated ELISPOT counter (AID Diagnostika GmbH, algorithm C), using identical settings for all plates and counts were adjusted only to remove artefacts. Responses to the negative control were always <80 SFC per million PBMC. Responses of >50 SFC per million after subtraction of background were considered positive.

For flow cytometry, responses were assessed by either a 7-colour (part A) or 12-colour (part B) staining panel, which was performed on freshly isolated PBMC, in parallel with ELISPOT assays, aliquots of 1 × 10^6^ cells in 1 ml of medium containing anti-CD28 and anti-CD49d at 1 μg ml^−1^ (Becton Dickinson) and 62.5 μg ml^−1^ of CD107a-PeCy5 (eBioscience)) in part B were stimulated with either no antigen, a pool of all 56 peptides spanning the T9/96 strain of the TRAP antigen (1 μg ml^−1^) or a positive control, Staphylococcal enterotoxin B (Sigma, 1 μg ml^−1^) in 5 ml polystyrene FACS tubes for 18 h. Brefeldin A and monensin, both at 1 μg ml^−1^, were added for the last 16 h. Cells were incubated with a dead cell discrimination dye (VIVID, 1/80, Invitrogen), and then surface-stained at 4°C with CD4-APC (1/20, eBioscience) or CD4-Qdot 625 (1/50, Invitrogen), CD14- and CD19-Pacific Blue (both 1/50, Becton Dickinson). After permeabilisation, intracellular staining was performed at room temperature with CD3-PeCy5 (part A, 1/20, eBioscience) or CD3-Alexa Fluor 700 (part B, 1/100, eBioscience) plus CD8-APC-Alexa Fluor 780 (1/50) and IFN-γ-FITC (1/50), IL-2-PE (1/100) and TNFα-Pe-Cy7 (1/50, all eBioscience) and fixed in 4% paraformaldehyde. Acquisition was performed on the day of staining on a BD LSRII; at least 500,000 events were collected per sample. Data was prepared and analysis performed using FlowJo v8.8.6 (Treestar Inc,), Pestle v1.6 and Spice v5.05 (Mario Roederer, Vaccine Research Centre, NIAID, NIH). Dead cells (Vivid^+^), monocytes (CD14^+^) and B cells (CD19^+^) were excluded from the analysis. A time gate was first evaluated, and then cells were gated on lymphocytes, singlets, live CD3^+^, CD8^+^ or CD4^+^ (excluding double-positives), and then IFNγ and combinations of markers. A sample gating strategy is provided in Supplementary Fig. S2. Responses to peptide were determined after subtraction of the response in the unstimulated control for each sample. Pie charts were created using absolute measures with a threshold of 0.001%. Mean fluorescence integrity (MFI) was calculated using the geometric mean of the cytokine-positive population and integrated MFI (iMFI) represents the integration of the frequency with the geometric mean of the cytokine-secreting population, giving a measure of total amount of cytokine production.

nAb titres to ChAd63 vector were determined as follows. One day prior to assay, GripTite 293 cells (Invitrogen) were seeded in 96-well plates (3 × 10^4^ cells/well). Heat-inactivated test sera were diluted fourfold from 1:9 to 1:2,304 in 10% fetal bovine sera (FBS) in DMEM and incubated 1:1 with ChAd63 expressing the secreted alkaline phosphatase (SEAP) gene (8 × 10^7^ vp ml^−1^) for 1 h at 37 °C. Serum and virus were then added to 293 cells in a volume of 200 μl in duplicate for 1 h, after which serum and virus were aspirated and replaced with fresh 10% FBS in DMEM. A virus-only control was included. After 22–26 h at 37 °C, 50 μl of medium was assayed for SEAP activity using a Phospha-Light TROPIX phosphatase assay (Applied Biosystems) in black assay plates and luminescence was measured after 45 min on a Thermo-Fisher Varioskan Flash Luminometer. Anti-vector neutralization titres were defined as the dilution of serum showing 50% reduction in SEAP activity, based on observed % inhibition values relative to SEAP activity from virus alone.

Anti-TRAP IgG ELISAs were performed in Nunc-Immuno Maxisorp 96-well plates (Thermo Scientific) coated with 1 μg ml^−1^ of TRAP protein in carbonate–bicarbonate coating buffer (Sigma) overnight at 4 °C. Plates were washed with PBS Tween and blocked with 1% BSA in PBS. Sera were diluted at a starting concentration of 1:100, added in duplicate and serially diluted threefold. Plates were incubated for 2 h at room temperature and then washed as before. Goat anti-human whole IgG conjugated to alkaline phosphatase (Sigma) was added for 1 h at room temperature. Following a final wash, plates were developed by adding p-nitrophenylphosphate at 1 mg ml^−1^ in diethanolamine buffer (Pierce). Optical density (OD) was read at 405 nm on an ELx800 microplate reader. Serum antibody endpoint titres were taken as the *x* axis intercept of the dilution curve at an absorbance value three s.d greater than the OD405 for serum before vaccination. A standard positive serum sample was included in each assay as a reference control.

PCR for blood parasite density after CHMI used blood samples filtered on custom 24-well Whatman VFE plates and washed with PBS to reduce white blood cell numbers. DNA extraction was performed on 0.5 ml of the filtered blood using Qiagen QIAamp Blood mini kit using a modified vacuum manifold protocol as follows: volumes of protease, lysis buffer and ethanol were 40 μl, 400 μl and 400 μl, respectively, and wash buffer volumes increased to 750 μl. The second wash buffer allowed to soak on the DNA purification columns for 2 min before the vacuum was applied. Columns were transferred to collection tubes and centrifuged at 13,000 r.p.m. for 4 min to dry. DNA was eluted with 50 μl sterile 10 mM Tris (pH 8.0), incubating for 1 min at room temperature before centrifugation (1 min, 8,000 r.p.m.) to collect the DNA sample. Quantitative PCR was performed on extracted DNA, amplifying a 133-bp product from the 18S ribosomal gene of *P. falciparum* in a TaqMan probe-based QPCR using the following primers: forward- 5′-GTAATTGGAATGATAGGAATTTACAAGGT-3′, reverse 5′-TCAACTACGAACGTTTTAACTGCAAC-3′ and a TaqMan probe 5′-FAM-AACAATTGGAGGGCAAG-NFQ-MGB-3′ (Applied Biosystems)^45^,^46^. Triplicate reactions of 25 μl were set up with 5 μl DNA template, 12.5 μl Universal PCR Master Mix, no Amperase UNG (Applied Biosystems), 1 μl forward primer, 1 μl reverse primer, 0.25 μl TaqMan Probe (all at 10 pmol μl^−1^) and 5.25 μl nuclease-free water. Reactions were run on an Applied Biosystems Step One Plus PCR System with an initial 10 min hot start activation at 95 °C, followed by 15 s denaturation at 95 °C and a 60 s annealing and extension at 60 °C for 45 cycles. Each plate contained a no template control and a standard curve made by serial dilutions of a plasmid containing the 133-bp PCR product that had been adjusted to give a parasites per ml equivalent (based on copies of 18S per parasite genome, blood sample volume extracted from and volume of DNA template used per reaction) of 10^5^, 10^4^, 10^3^, 3 × 10^2^, 10^2^, 4 × 10^1^ and 2 × 10^1^. Automatic quantitation was performed with Applied Biosystems Step One plus software v2.1. Mean parasite equivalent values of <20 or with only 1 positive replicate were classed as negative. Laboratory staff performing blood film microscopy and qPCR were blinded to the group allocation of volunteers.

### Data analysis and statistics

Time to parasitaemia as measured by thick-film microscopy was the primary efficacy endpoint and this was analysed using Kaplan–Meier log-rank test. Assessments of efficacy compared unvaccinated controls with groups of immunized volunteers. Immunological analysis compared markers of T-cell immunogenicity (summed *ex vivo* and cultured ELISPOT responses, and percentage of antigen-specific CD4+ and CD8+ cells by intracellular cytokine staining) and antibody responses between groups. Group averages (mean or median) were described. Significance testing of differences between group means (for normally distributed data as assessed by the D’Agostino and Pearson omnibus normality test) used the two-tailed Student’s *t*-test, or medians used the two-tailed Mann–Whitney *U*-test. Immunological correlations with time to patent parasitaemia or other variables were pre-specified in the trial protocol as secondary endpoints and prioritized analysis of T-cell subsets (particularly CD8^+^ subpopulations), based on observations from pre-clinical studies with these vaccines. Analyses were performed using the two-tailed Spearman’s rank correlation co-efficient *(r*_s_) as data were either non-normally distributed or the number of observations was too small to allow an accurate estimation of the underlying distribution. Correction of *P*-values for multiple comparisons was made using the Bonferroni method, which multiplies the *P* value by the number of comparisons assessed. For log-transformed PCR data, an arbitrary value of 1 was added to each PCR value to allow log transformation of 0.0 values (*y*=log (1+PCR)). The area under curves was calculated using the trapezoid rule and compared between groups as described above. Regression models were fitted to log-transformed flow cytometry data with frequency of given T-cell populations as the independent variable and time to parasitaemia as the dependent variable, and then Akaike’s information criterion was used as an aid for choosing between competing models. Lower values of the index indicate the preferred model, that is, the one with the fewest parameters that still provides an adequate fit to the data. An alpha level of 0.05 was considered statistically significant. Statistical analysis was performed using Prism 5.0, Graph Pad Software.

### Additional clinical information

Over 300 individuals had previously participated in controlled human malaria infections by mosquito bite at the Oxford centre and over a 1,000 further volunteers worldwide, all as part of vaccine studies. The clinical trial methodologies have been found to be safe and have been reviewed recently^47^,^48^. In this trial volunteers received as compensation sums ranging from £1,500 to £1,800, varying according to the number of clinic visits made.

## Author contributions

Designed the study and associated protocols: K.J.E., G.A.O.H., C.J.A.D., A.L.G., A.R.-S., R.E.S., R.C., S.C.G., A.N. and A.V.S.H.; undertook research work: K.J.E., G.A.O.H., C.J.A.D., K.A.C., S.H.S., A.L.G., N.J.E., S.C.E., F.D.H., R.J.L., R.R., I.D.P., S.J.D., A.M.B., E.B., S.M., S.C., L.S., A.F. and A.M.L.; analysed data: K.J.E., G.A.O.H., C.J.A.D., K.A.C., R.J.L., N.W., S.C.G., A.N. and A.V.S.H.; wrote the manuscript: K.J.E., G.A.O.H., C.J.A.D. and A.V.S.H.

## Additional information

**How to cite this article:** Ewer, K. J. *et al.* Protective CD8^+^ T-cell immunity to human malaria induced by chimpanzee adenovirus-MVA immunization. *Nat. Commun.* 4:2836 doi: 10.1038/ncomms3836 (2013).

## Supplementary Material

Supplementary Information Supplementary Figures S1-S3, Supplementary Tables S1-S7 and Supplementary Methods

## Figures and Tables

**Table 1:** Analysis of potential immune correlates with time to parasitaemia.

**Assay**	**Population/response**	***N***	**Time point**	***r***	***P*****-value**
TRAP ELISA	IgG antibody titre	18	Challenge A	0.16	0.536
Functional Assay	Neutralizing antibodies to ChAd63	24	Screening/Day 0Challenges A+B	0.13	0.534
*Ex vivo* ELISPOT	SFC’s/10^6^ PBMC (TRAP peptides)	24	Challenges A+B	0.27	0.203
Cultured ELISPOT	SFC’s/10^6^ PBMC (TRAP peptides)	18	Challenge A	0.39	0.109
Flow cytometry	CD4^+^ IFNγ^+^	18	Challenge A	0.46	0.056
	CD4^+^ IFNγ^+^	14 (Ad-MVA)	Challenges A+B	0.3	0.3
	CD4^+^ IL-2^+^	18	Challenge A	0.53	0.025*
	CD4^+^ IL-2^+^	14 (Ad-MVA)	Challenges A+B	0.23	0.44
	CD8^+^ IFNγ^+^	18	Challenge A	0.57	0.013*
	CD8^+^ IFNγ^+^	14 (Ad-MVA)	Challenges A+B	0.45	0.11
	CD8^+^ IL-2^+^	18	Challenge A	0.55	0.019*
	CD8^+^ IL-2^+^	14 (Ad-MVA)	Challenges A+B	0.16	0.58
	CD8^+^ TNFα^+^	18	Challenge A	0.51	0.030*
	CD8^+^ TNFα^+^	14 (Ad-MVA)	Challenges A+B	0.07	0.82
	CD8^+^ IFNγ^+^ (iMFI)	18	Challenge A	0.55	0.017*
	CD8^+^ IFNγ^+^ (iMFI)	14 (Ad-MVA)	Challenges A+B	0.27	0.35
	CD4^+^ IFNγ^+^/IL-2^+^/TNFα^+^	18	Challenge A	0.45	0.059
	CD4^+^ IFNγ^+^/IL-2^+^/TNFα^+^	14 (Ad-MVA)	Challenges A+B	0.26	0.37
	CD8^+^ IFNγ^+^/IL-2^+^/TNFα^+^	18	Challenge A	0.47	0.052
	CD8^+^ IFNγ^+^/IL-2^+^/TNFα^+^	14 (Ad-MVA)	Challenges A+B	0.1	0.73
	CD8^+^ IFNγ^+^/IL-2^−^/TNFα^−^	18	Challenge A	0.63	0.005*
	CD8^+^ IFNγ^+^/IL-2^−^/TNFα^−^	8 (Ad-MVA)	Challenge A	0.84	0.011*
	CD8^+^ IFNγ^+^/IL-2^−^/TNFα^−^ MFI	18	Challenge A	0.44	0.067
	CD8^+^ IFNγ^+^/IL-2^−^/TNFα^−^ iMFI	18	Challenge A	0.55	0.017*
	CD8^+^ IFNγ^+^/IL-2^−^/TNFα^−^ iMFI	14 (Ad-MVA)	Challenges A+B	0.46	0.1
	CD8^+^ CD107a^+^/IFNγ^−^/IL-2^−^/TNFα^−^	14	Challenges A+150	0.61	0.020*
	CD8^+^ IFNγ^+^/IL-2^−^/TNFα^−^	6 (Ad-MVA)	Challenge B	0.64	0.018*
	CD8^+^ IFNγ^+^/IL-2^−^/TNFα^−^	24	Challenges A+B	0.61	0.0016*
	CD8^+^ IFNγ^+^/IL-2^−^/TNFα^−^	14 (Ad-MVA)	Challenges A+B	0.81	0.0005†

Correlation coefficients between time to parasitaemia and different immune measures (two-tailed Spearman’s correlation). A total of 18 individuals (10 administered Ad alone and 8 Ad-MVA) were included from challenge A and 6 (all administered Ad-MVA) from challenge B.

Regression models were fitted to the log-transformed data using either frequency of CD4^+^ IFNγ^+^, CD8^+^ IFNγ^+^ or both T cell populations as the independent variable and time to parasitaemia as the dependent variable and Akaike’s information criterion (AIC) was calculated for each. The best fitting model was CD8^+^ IFNγ^+^ (AIC 126.6) and the model with CD4^+^ IFNγ^+^ alone as the independent variable fitted least well (AIC 130.4). Adding CD4^+^ IFNγ^+^ to the CD8^+^ IFNγ^+^ model did not improve the fit significantly (likelihood ratio test, *P*=0.77). MFI and iMFI relate to IFNγ staining.

^*^Statistically significant at *P*<0.05.

^†^Correction of this *P*-value by a factor of 10 to make a conservative Bonferroni correction for multiple comparisons yields a *P*-value of *P*_corrected_=0.005.
