# Supplementary material for: Protective CD8+ T-cell immunity to human malaria induced by chimpanzee adenovirus-MVA immunisation
Source: Nat Commun. 2013 Nov 28;4:2836. doi: 10.1038/ncomms3836 (PMC3868203; doi:10.1038/ncomms3836)
Supplement: Supplementary Information — Supplementary Figures S1-S3, Supplementary Tables S1-S7 and Supplementary Methods [file ncomms3836-s1.pdf]

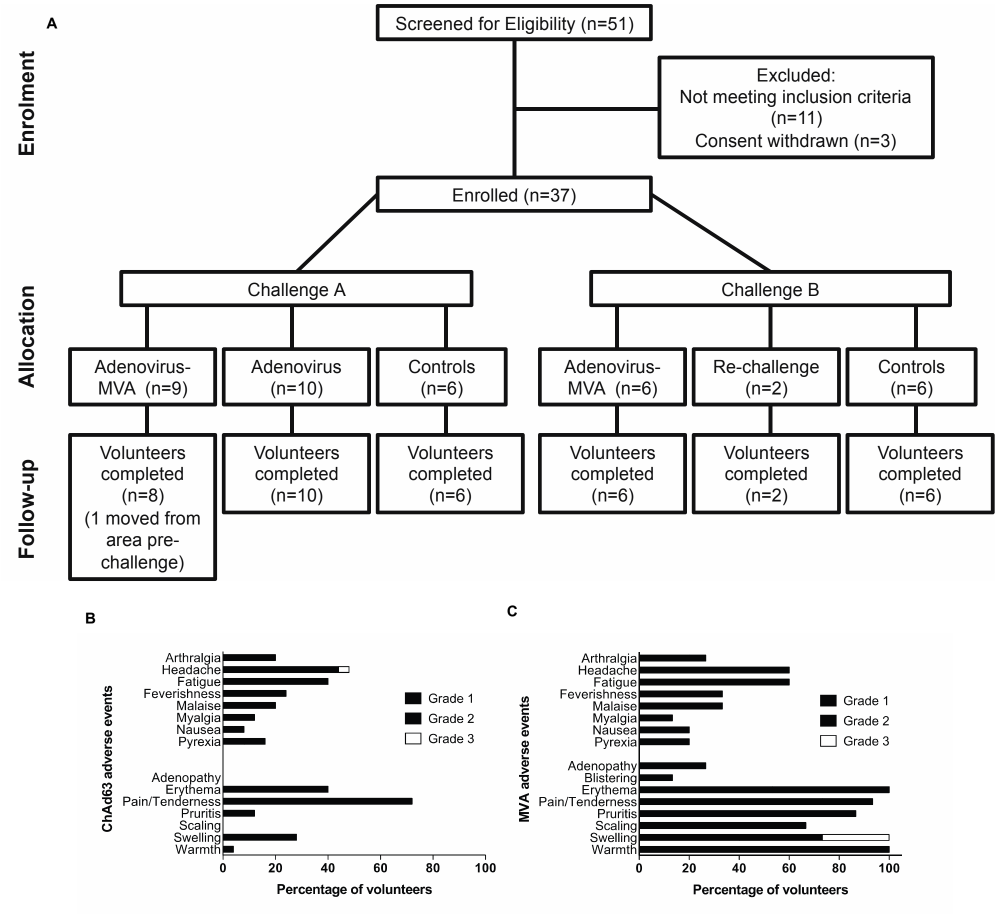

Supplementary Fig. S1. (A) Volunteers were challenged in two parts. Challenge A: eighteen vaccinees and six unvaccinated controls. Challenge B: six vaccinees and six unvaccinated controls, with re-challenge of two protected volunteers from challenge A. The one protected volunteer from challenge B was re-challenged in a subsequent trial 8 months later. Adenovirus (B) and MVA (C) related systemic and injection-site reactions by grade up to 28 days. There were no immunization or challenge related serious adverse events. One volunteer experienced grade 1 neutropenia 7 days post MVA, which resolved on repeat testing. Three volunteers experienced grade 1 neutropenia (one Ad-M, one Ad, one control) and one control volunteer had transient grade 1 thrombocytopenia post-challenge, which resolved on repeat testing.

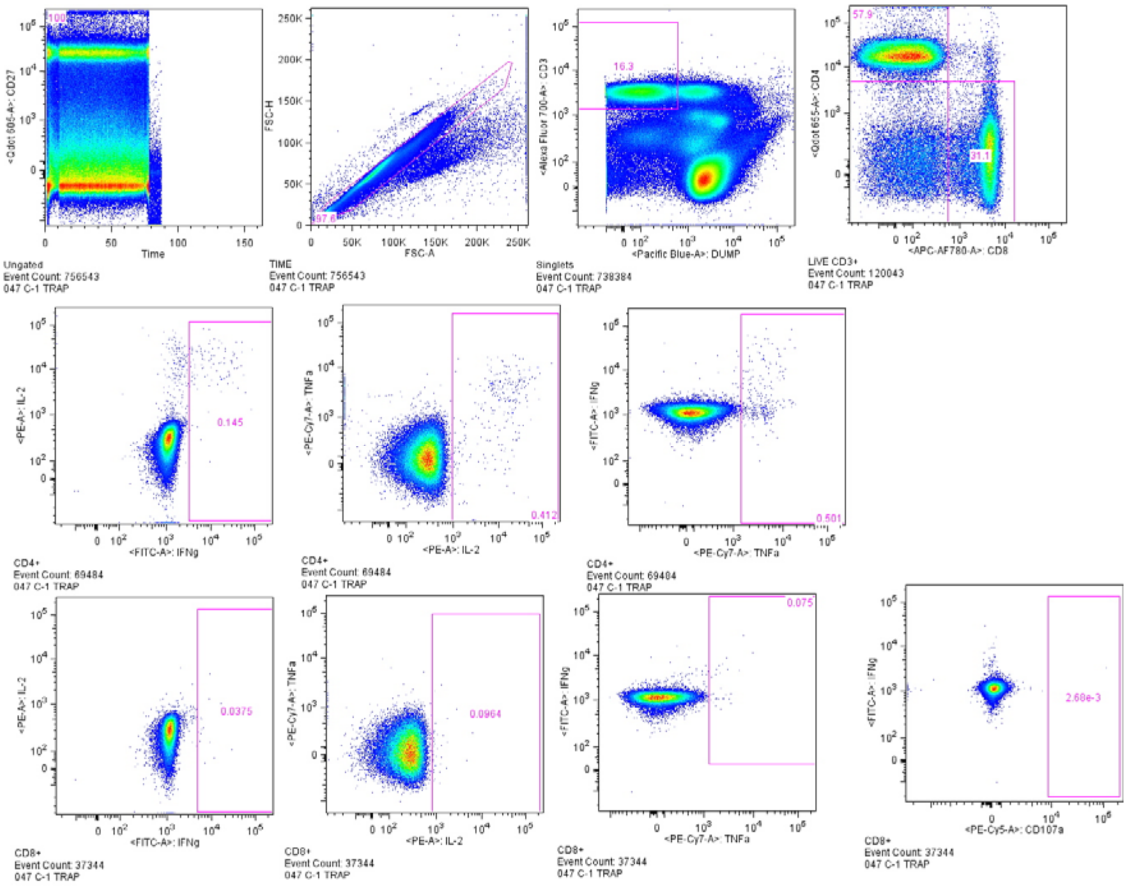

Supplementary Fig. S2. Flow cytometry gating strategy. Singlets were identified using forward scatter plots. Dead cells were excluded by violet fluorescent amine-reactive dye staining. Monocytes and B cells were excluded by CD14 or CD19 expression and T cells identified by CD3 expression. T cells were then subdivided by gating on $CD4^+$ and $CD8^+$ populations. Cytokine expression was quantified by plotting pairs of cytokines against each other and gating positive populations. This is a representative sample from a volunteer in the Ad-M group stimulated overnight (18 hours) with a single pool of overlapping TRAP peptides.

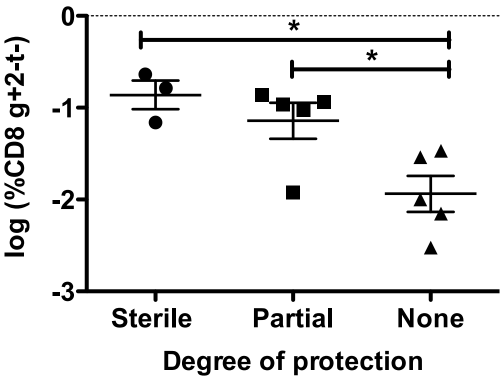

Supplementary Fig. S3. Frequencies of $CD8^{+}$ $IFN\gamma^{+}IL2^{-}TNF\alpha^{-}$ T cells before challenge (means +/-SEM), p=0.009 across groups using 1 way ANOVA. * P<0.05 after Bonferroni multiple comparison test for comparison between groups.

Supplementary Table S1. PCR data

Group	Challenge	6.5	7	7.5	8	8.5	9	9.5	10	10.5	11	11.5	12
**Ad-M**	A	N	N	56	57	32							
**Ad-M**	A	N	N	N	N	N	N	N	N	N	59	N	560
**Ad-M**	A	N	N	N	N	N	N	N	N	N	N	N	N
**Ad-M**	A	N	N	29	N	N	31	52	N	89	855	1172	440
**Ad-M**	A	N	N	N	N	N	N	N	N	N	N	N	N
**Ad-M**	A	N	N	N	N	N	N	N	64	85			
**.Ad-M**	A	N	N	66	54	N	261	173	72	504	520	1277	
**Ad-M**	A	N	N	30	N	N	21	116	N	23	462	385	252
**Ad-M**	B	N	N	N	N	N	N	N	N	N	30	20	N
**Ad-M**	B	N	N	N	N	N	N	N	N	N	N	N	N
**Ad-M**	B	N	38	105	56	44	147	187	36	126	629	619	1764
**Ad-M**	B	N	N	N	N	N	N	N	N	N	N	N	25
**Ad-M**	B	N	N	N	N	N	37	50	N	26	509	169	236
**Ad-M**	B	N	N	N	34	N	25	24	N	N	450	66	91
**Ad**	A	N	43	223	179	119	663	1214	521	3038	11223		
**Ad**	A	N	N	N	N	N	N	N	N	42	63	30	560
**Ad**	A	N	186	235	293	208	1634	2272					
**Ad**	A	N	N	88	N	N	52	175	31	282	940	690	914
**Ad**	A	N	N	N	20	N	41	82	N	267	**		
**Ad**	A	N	98	248	146	34	1192	1784	214	3291	8947	4531	
**Ad**	A	N	N	N	N	N	26	N	N	75	96	46	932
**Ad**	A	N	70	53	33	N	278	352	93	842	5707		
**Ad**	A	N	N	169	79	74							
**Ad**	A	N	N	37	43	N	46	131	N	119	**		
**C**	A	N	N	N	N	N	N	N	N	N	516	268	212
**C**	A	N	N	48	34	27	189						
**C**	A	N	N	42	47	N	73	177	58	105	985	1737	1563
**C**	A	N	N	N	73	N	426	291	232	281	1526	3139	1772
**C**	A	N	N	N	N	N	35	28	27	372	283	288	5878
**C**	A	N	N	29	N	N	27	64	79	156	194	446	1300
**C**	B	N	51	154	60	50	464	968	230	1129	7885		
**C**	B	N	N	N	N	N	27	24	23	104	276	124	2078
**C**	B	N	N	N	N	27	N	39	34	89	420	336	1486
**C**	B	N	N	39	N	N	90						
**C**	B	N	76	177	206	56	383	336	417	1190	4596	2530	25195
**C**	B	N	N	33	32	53	73	272	433	1464	5007		
**Re-Ch**	B	N	N	N	N	N	N	N	N	24	20	N	485
**Re-Ch**	B	N	N	N	N	N	N	N	N	N	N	N	N

Group	Challenge	12.5	13	13.5	14	14.5	15	16	17	18	19	20	21
**Ad-M**	A												

**Ad-M**	A	2058	291	37	20087								
**Ad-M**	A	N	N	N	N	N	N	N	N	N	N	N	N
**Ad-M**	A	3294	5983	1601	37048								
**Ad-M**	A	N	N	N	N	N	N	N	N	N	N	N	N
**Ad-M**	A												
**Ad-M**	A												
**Ad-M**	A	3951											
**Ad-M**	B	226	125	N	2894	4196	318						
**Ad-M**	B	N	N	N	N	N	N	N	N	N	N	N	N
**Ad-M**	B	13046											
**Ad-M**	B	22	N	393	486	86	2773						
**Ad-M**	B	2157											
**Ad-M**	B	749	1790	850	8135	11923							
**Ad**	A												
**Ad**	A	1531	778	2618	24389								
**Ad**	A												
**Ad**	A	9238	4475										
**Ad**	A												
**Ad**	A												
**Ad**	A	2750	1312										
**Ad**	A												
**Ad**	A												
**Ad**	A												
**C**	A	3248	4032	442	82315								
**C**	A												
**C**	A	11178											
**C**	A												
**C**	A	22779											
**C**	A	3816	3170										
**C**	B												
**C**	B	4189											
**C**	B	2199											
**C**	B												
**C**	B												
**C**	B												
**Re-Ch**	B	942	112	1379	12013								
**Re-Ch**	B	N	N	N	N	N	N	N	N	N	N	N	N

Supplementary Table S1. Raw qPCR data (parasites/ml). Top row represents day of follow-up visit post challenge. Day of diagnosis is represented by grey highlighting. Controls were diagnosed on days 9, 9, 11, 11, 12, 12, 12.5, 12.5, 12.5, 12.5, 13 and 14. N = negative (i.e. <20 parasites/ml).

Ad-M = ChAd63 ME-TRAP with MVA ME-TRAP. Ad = ChAd63 ME-TRAP alone. C = unvaccinated controls. Re-Ch = Re-challenge of sterilely protected volunteers from challenge A. ** sample not analysed.

Supplementary Table S2. TRAP peptide pool format.

Peptide Name	Peptide sequence T9/96	Peptide sequence 3D7	T9/96 Peptide Pool	3D7 Peptide Pool
TRAP-1	MNHLGNVKYLVIVFLIFFDL		TT1-10	TD1-10
TRAP-2	VIVFLIFFDLFLVNGRDVQN		TT1-10	TD1-10
TRAP-3	FLVNGRDVQNNIVDEIKYSE	**FLVNGRDVQNNIVDEIKYRE**	TT1-10	TD1-10
TRAP-4	NIVDEIKYSEEVCNDQVDLY	**NIVDEIKYREEVCNDEVDLY**	TT1-10	TD1-10
TRAP-5	EVCNDQVDLYLLMDCSGSIR	**EVCNDEVDLYLLMDCSGSIR**	TT1-10	TD1-10
TRAP-6	LLMDCSGSIRRHNWVNHAVP		TT1-10	TD1-10
TRAP-7	RHNWVNHAVPLAMKLIQQLN		TT1-10	TD1-10
TRAP-8	LAMKLIQQLNLNDNAIHLYV	**LAMKLIQQLNLNDNAIHLYA**	TT1-10	TD1-10
TRAP-9	LNDNAIHLYVNVFSNNAKEI	**LNDNAIHLYASVFSNNAREI**	TT1-10	TD1-10
TRAP-10	LNDNAIHLYVNVFSNNAKEI	**SVFSNNAREIIRLHSDASKN**	TT1-10	TD1-10
TRAP-11	IRLHSDASKNKEKALIIIRS	**IRLHSDASKNKEKALIIIKS**	TT11-20	TD11-20
TRAP-12	KEKALIIIRSLLSTNLPYGR	**KEKALIIIKSLLSTNLPYGK**	TT11-20	TD11-20
TRAP-13	LLSTNLPYGRTNLTDALLQV	**LLSTNLPYGKTNLTDALLQV**	TT11-20	TD11-20
TRAP-14	TNLTDALLQVRKHLNDRINR		TT11-20	TD11-20
TRAP-15	RKHLNDRINRENANQLVVIL		TT11-20	TD11-20
TRAP-16	ENANQLVVILTDGIPDSIQD		TT11-20	TD11-20
TRAP-17	TDGIPDSIQDSLKESRKLSD		TT11-20	TD11-20
TRAP-18	SLKESRKLSDRGVKIAVFGI		TT11-20	TD11-20
TRAP-19	RGVKIAVFGIGQGINVAFNR		TT11-20	TD11-20
TRAP-20	GQGINVAFNRFLVGCHPSDG		TT11-20	TD11-20
TRAP-21	FLVGCHPSDGKCNLYADSAW		TT21-30	TD21-30
TRAP-22	KCNLYADSAWENVKNVIGPF		TT21-30	TD21-30
TRAP-23	ENVKNVIGPFMKAVCVEVEK		TT21-30	TD21-30
TRAP-24	MKAVCVEVEKTASCGVWDEW		TT21-30	TD21-30
TRAP-25	TASCGVWDEWSPCSVTCGKG		TT21-30	TD21-30
TRAP-26	SPCSVTCGKGTRSRKREILH		TT21-30	TD21-30
TRAP-27	TRSRKREILHEGCTSEIQEQ	**TRSRKREILHEGCTSELQEQ**	TT21-30	TD21-30
TRAP-28	EGCTSEIQEQCEEERCPPKW	**EGCTSELQEQCEEERCLPKR**	TT21-30	TD21-30
TRAP-29	CEEERCPPKWEPLDVPDEPE	**CEEERCLPKREPLDVPDEPE**	TT21-30	TD21-30
TRAP-30	EPLDVPDEPEDDQPRPRGDN		TT21-30	TD21-30
TRAP-31	DDQPRPRGDNSSVQKPEENI	**DDQPRPRGDNFAVEKPNENI**	TT31-40	TD31-40
TRAP-32	SSVQKPEENIIDNNPQEPSP	**FAVEKPNENIIDNNPQEPSP**	TT31-40	TD31-40
TRAP-33	IDNNPQEPSPNPEEGKDENP	**IDNNPQEPSPNPEEGKGENP**	TT31-40	TD31-40
TRAP-34	NPEEGKDENPNGFDLDENPE	**NPEEGKGENPNGFDLDENPE**	TT31-40	TD31-40
TRAP-35	NGFDLDENPENPPNPDIPEQ	**NGFDLDENPENPPNPPNPPN**	TT31-40	TD31-40
TRAP-36	NPPNPDIPEQKPNIPEDSEK	**NPPNPPNPPNPPNPPNPPNP**	TT31-40	TD31-40
TRAP-37	*NONE*	**PPNPPNPPNPDIPEQKPNIP**	TT31-40	TD31-40
TRAP-38	DIPEQKPNIPEDSEKEVPSD	**DIPEQKPNIPEDSEKEVPSD**	TT31-40	TD31-40
TRAP-39	EDSEKEVPSDVPKNPEDDRE		TT31-40	TD31-40
TRAP-40	VPKNPEDDREENFDIPKKPE		TT31-40	TD31-40
TRAP-41	ENFDIPKKPENKHDNQNNLP		TT41-50	TD41-50
TRAP-42	NKHDNQNNLPNDKSDRNIPY	**NKHDNQNNLPNDKSDRYIPY**	TT41-50	TD41-50
TRAP-43	NDKSDRNIPYSPLPPKVLDN	**NDKSDRYIPYSPLAPKVLDN**	TT41-50	TD41-50
TRAP-44	SPLPPKVLDNERKQSDPQSQ	**SPLAPKVLDNERKQSDPQSQ**	TT41-50	TD41-50
TRAP-45	ERKQSDPQSQDNNGNRHVPN		TT41-50	TD41-50
TRAP-46	DNNGNRHVPNSEDRETRPHG		TT41-50	TD41-50
TRAP-47	SEDRETRPHGRNNENRSYNR		TT41-50	TD41-50
TRAP-48	RNNENRSYNRKYNDTPKHPE		TT41-50	TD41-50
TRAP-49	KYNDTPKHPEREEHEKPDNN		TT41-50	TD41-50
TRAP-50	REEHEKPDNNKKKGESDNKY		TT41-50	TD41-50
TRAP-51	KKKGESDNKYKIAGGIAGGL		TT51-57	TT51-57

TRAP-52	KIAGGIAGGLALLACAGLAY		TT51-57	TT51-57
TRAP-53	ALLACAGLAYKFVVPGAATP		TT51-57	TT51-57
TRAP-54	KFVVPGAATPYAGEPAPFDE		TT51-57	TT51-57
TRAP-55	YAGEPAPFDETLGEEDKDLD		TT51-57	TT51-57
TRAP-56	TLGEEDKDLDEPEQFRLPEE		TT51-57	TT51-57
TRAP-57	EPEQFRLPEENEWN		TT51-57	TT51-57

Supplementary Table S3. ME peptide pool format.

Peptide Name	Peptide sequence	Antigen
st8	MINAYLDKL	STARP
ls50	ISKYEDEI	LSA1
pb9	SYIPSAEKI	*P. berghei CSP*
ls8	KPNDKSLY	LSA1
cp26	KPKDELDY	CSP
ls6	KPIVQYDNF	LSA1
tr42/43	ASKNKEKALII	TRAP
tr39	GIAGGLALL	TRAP
cp6	MNPNDPNRNV	CSP
tr26	HLGNVKYLV	TRAP
ls53	KSLYDEHI	LSA1
tr29	LLMDCSGSI	TRAP
csp	DPNANPNVDPNANPNV	CSP
38H BCG	QVHFQPLPPAVVKL	BCG
FTTp	QFIKANSKFIGITE	TT
cp39	YLNKIQNSL	CSP
la72	MEKLKELEK	LSA3
ex23	ATSVLAGL	EXP1
nanp	NANPNANPNANPNANP	CSP
trapAM	DEWSPCSVTCGKGTRSRKRE	TRAP

Supplementary Table S4. Criteria for assessment of systemic adverse event severity.

Grade	Criteria
0	None
1	Mild: Transient or mild discomfort (< 48 hours); no medical intervention/therapy required
2	Moderate: Mild to moderate limitation in activity - some assistance may be needed; no or minimal medical intervention/therapy required
3	Severe: Marked limitation in activity, some assistance usually required; medical intervention/therapy required, hospitalisation possible
4	Serious, life-threatening: Extreme limitation in activity, significant assistance required; significant medical intervention/therapy required, hospitalisation or hospice care probable.

Supplementary Table S5. Specific criteria for severity grading of injection site reactions.

	**Grade**	**Diameter [mm] / Description**
**Swelling**		
	0	0
	1	< 20
	2	20 – 50
	3	> 50
**Erythema**		
	0	0
	1	< 50
	2	50 – 100
	3	> 100
**Pain**		
	**0**	No pain at all
	**1**	Painful to touch, no restriction in movement of arms, able to work,drive, carry heavy objects as normal
	**2**	Painful when limb is moved (*i.e.* restriction in range of movement in arm, difficulty in carrying objects)
	**3**	Severe pain at rest (*i.e.* unable to use arm due to pain.)

Supplementary Table S6. Assessment of adverse event outcome

1	Recovered
2	Recovered with sequelae
3	Died
4	Unknown
5	On-going at subject study conclusion (active phase)

Supplementary Table S7. Assessment of adverse event relatedness to vaccination.

0	**No Relationship**	No temporal relationship to study product ***and***<br>Alternate aetiology (clinical state, environmental or other interventions); ***and***<br>Does not follow known pattern of response to study product
1	**Possible**	Reasonable temporal relationship to study product; ***or***<br>Event not readily produced by clinical state, environmental or other interventions; ***or***<br>Similar pattern of response to that seen with other vaccines
2	**Probable**	Reasonable temporal relationship to study product; ***and***<br>Event not readily produced by clinical state, environment, or other interventions ***or***<br>Known pattern of response seen with other vaccines
3	**Definite**	Reasonable temporal relationship to study product; ***and***<br>Event not readily produced by clinical state, environment, or other interventions; ***and***<br>Known pattern of response seen with other vaccines

Supplementary Methods-Clinical inclusion and exclusion criteria

Inclusion Criteria

The volunteer must satisfy all the following criteria to be eligible for the study:

- Healthy adult aged 18 to 50 years
- Able and willing (in the Investigator's opinion) to comply with all study requirements
- Willing to allow the investigators to discuss the volunteer's medical history with their General Practitioner
- For females only: willingness to practise effective contraception throughout the study
- Agreement to refrain from blood donation during the course of the study
- Written informed consent

Exclusion Criteria

The volunteer may not enter the study if any of the following apply:

- Participation in another research study involving an investigational product in the 30 days preceding enrolment, or planned use during the study period.
- Prior receipt of an investigational malaria vaccine encoding ME-TRAP or any other investigational vaccine likely to impact on interpretation of the trial data
- Administration of immunoglobulins and/or any blood products within the three months preceding the planned administration of the vaccine candidate
- Any confirmed or suspected immunosuppressive or immunodeficient state, including HIV infection; asplenia; recurrent, severe infections and chronic (more than 14 days) immunosuppressant medication within the past 6 months (inhaled and topical steroids are allowed)
- Pregnancy, lactation or intention to become pregnant during the study
- Contraindication to both anti-malarial drugs (Riamet® and chloroquine)

- Concomitant use with other drugs known to cause QT-interval prolongation, (e.g. macrolides, quinolones, amiodarone etc.)
- An estimated, ten year risk of fatal cardiovascular disease of $\geq$5%, as estimated by the Systematic Coronary Risk Evaluation (SCORE) system
- History of arrhythmia or prolonged QT interval;
- Positive family history for sudden cardiac death
- History of allergic disease or reactions likely to be exacerbated by any component of the vaccine, e.g. egg products, Kathon.
- History of clinically significant contact dermatitis
- Any history of anaphylaxis in reaction to vaccination
- History of cancer (except basal cell carcinoma of the skin and cervical carcinoma in situ)
- History of serious psychiatric condition
- Any other serious chronic illness requiring hospital specialist supervision
- Suspected or known current alcohol abuse as defined by an alcohol intake of greater than 42 units every week
- Suspected or known injecting drug abuse
- Seropositive for hepatitis B surface antigen (HBsAg)
- Seropositive for hepatitis C virus (antibodies to HCV)
- Any other significant disease, disorder or finding, which, in the opinion of the Investigator, may either put the volunteer at risk because of participation in the study, or may influence the result of the study, or the volunteer's ability to participate in the study.
- History of clinical *P. falciparum* malaria
- Travel to a malaria endemic region during the study period or within the previous six months
- Any clinically significant abnormal finding on screening biochemistry or haematology blood tests or urinalysis

- Any other finding which in the opinion of the investigators would significantly increase the risk of having an adverse outcome from participating in the protocol or impair interpretation of the study data.
- In Challenge A neutralising antibody titres to ChAd63 of greater than 200 was an exclusion criterion for vaccination, however this was removed for vaccinated volunteers in Challenge B.
